# Association between carotid area calcifications and periodontal risk: a cross sectional study of panoramic radiographic findings

**DOI:** 10.1186/1471-2261-11-67

**Published:** 2011-11-09

**Authors:** Ralph Tiller, Wolfgang Bengel, Sven Rinke, Dirk Ziebolz

**Affiliations:** 1 Private Dental Practice, Hamburg, Germany; 2 Private Dental Practice, Heiligenberg, Germany; 3 Department of Preventive Dentistry, Periodontology and Cariology, University Medical Center Goettingen, Germany

## Abstract

**Background:**

The aim was to investigate the extent to which it is possible to diagnose suspected carotid calcification from dental panoramic radiography (PR) and to establish an association to periodontal risk.

**Methods:**

824 PRs from one dental practice were investigated. Parameters considered were gender, age, bone loss - age index, tooth loss, periodontal risk and suspected carotid calcification (left, right, both sides). Periodontal risk was classified: low risk (under 4 missing teeth, bone loss - age index under 0.5), moderate risk (5 to 8 missing teeth and/or bone loss - age index 0.5 to 1.0) and high risk (more than 9 missing teeth and or bone loss - age index greater than 1.0).

**Results:**

Of 824 patients, 349 were male (42.4%) and 475 female (57.6%); the mean age was 48.32 ± 16.52 years. In 9.0% (n = 74) of PRs, suspected carotid calcification was diagnosed (right: 5.5%, left: 2.3%, both sides: 1.2%). The mean tooth loss was 4.16 ± 5.39 teeth. In the case of 282 patients (34.2%), there was a low, in 335 patients (40.7%) a moderate, and in 207 patients (25.1%) a high periodontal risk. There was a significant correlation found between number of cases of suspected carotid calcification and periodontal risk, tooth loss and age (p = 0.0001). However, only age showed a significant association (OR: 4.9; CI: 2.4-9.8; p < 0.0001) in contrast to periodontal risk (OR 1.4; CI: 0.9-2.4).

**Conclusion:**

PR can provides indication of carotid calcification as a secondary (chance) finding. In addition, periodontal risk may be correlated with positive findings of carotid calcification.

## Background

Cardiovascular diseases such as coronary heart disease (CHD) and apoplexy, as well as cerebrovascular infarct, are amongst the most frequent causes of death in industrialized countries [1,2]. Also, some cerebrovascular insults are preceded by stenosis and/or calcification of the carotid artery [3]. Moreover, diagnosed calcification of the carotid artery can be a sign of additional calcification of the coronary arteries [3].

Early recognition of such calcifications could therefore minimize the risk of a subsequent stroke or coronary heart disease (CHD). Until now, sonography has been considered to be the "gold standard", amongst non-invasive techniques used to establish arteriosclerotic changes in the extra-cranial vessels, such as the carotid artery [4]. However, ultrasound investigations are not routinely carried out as a preventative measure in all patients. More time-consuming methods of diagnosing calcification, such as computer tomography (CT), magnet resonance tomography (MRT) and electronic thermography (ET), as well as the invasive method of angiography, have special indications and are not routinely used for all patients [5]. Accordingly, alternative diagnostic aids to enable quick and economic investigations to be carried out are needed. A number of authors have commented on the importance and possibilities of diagnostics for calcification of the carotid artery using panoramic radiography (PR) [6,7]. This suggests that imaging of calcified plaque of the carotid artery using dental PR is possible with a high degree of reliability [7-12]. The probability interval of finding carotid calcification in a PR lies between 2.5% and 4.1% [5].

The thought of using dental PR as an early and, above all, economic diagnostic tool for detecting calcification of the carotid artery is certainly worth considering. After all, a large number of patients regularly go for dental check-ups and do so more frequently than undergoing routine health checks. The PR is a basic diagnostic procedure in dental investigations. Calcification can be detected and documented as a secondary (chance) finding in PRs. If abnormal findings are established during the procedure, then a more precise diagnostic is to be recommended and initiated by specialists in internal medicine.

The oral cavity is a point of entry into the blood circulation for microorganisms. Therefore, inflammations in the area of the oral cavity such as, for example, periodontitis with accompanying highly pathogenic bacteria, in the sense of a chronic-relapsing bacteremia occurring over a long period of time and distributed throughout the whole body, can have a direct effect on the general health of the patient. A number of studies have described a corresponding association with different systemic diseases such as, for example, diabetes mellitus and premature births, but also cardiovascular diseases, myocardial infarction, as well as arteriosclerosis and strokes [13-22]. The stronger the oral infection is, the greater is the risk of a bacteremia [23,24], and consequently the risk of systemic diseases [13]. Above all, the correlation between periodontal disease and arteriosclerosis has been attributed particular importance [18,19,25]. Several systematic reviews and meta-analyses have assessed an evidence regarding an association between periodontal disease and cardiovascular diseases, e.g. arteriosclerosis and/or carotid calcification [18,26-29].

The aim of this study was to investigate, in a dental practice, the extent to which the suspicion (prevalence) of carotid calcification can be diagnosed as a secondary (chance) finding in dental PRs and which factors: age, gender, number of missing teeth and periodontal risk are correlated with the occurrence of carotid calcification, and to establish an association to periodontal risk. In this context, it was the intention to determine what value for health the appropriate diagnostics could have based on dental PR in everyday practice.

## Methods

This study was a cross-sectional study based on exclusive available panoramic radiographs (PR) from one specific private dental practice in Germany. The study was reviewed and approved by the ethics committee of the University Medical Center Goettingen, Germany (No. 9/10/08).

### Panoramic radiograph (PR)

Only dental PRs generated between 1984 and 2009 were evaluated in this investigation (n = 1521). All PRs were performed with the orthopantomograph (x-ray machine) OP 10 E (Siemens, Bensheim, Germany). The x-ray parameters were for women 75 kV, 6 mA, 15 sec. and for men 75 kV, 10 mA, 15 sec; the position of the patients in the orthopantomograph was individually adjusted. The PR images were prepared manually according to a pre-established and standardized protocol on conventional films by one and the same skilled operator (dental assistant).

PR pictures that showed defects resulting from the imaging process or the area of interest for carotid calcification could not be observed sufficiently, respectively, were sorted out in advance and were not considered in the investigation (n = 56/4%). Furthermore, in case there was more than one PR of the same patient only the most recent PR was evaluated. On the evaluted PRs no identifiers were present.

### Data evaluation

The selected PR pictures were used to produce the patient-related data from the documented treatment documents. Amongst others, the following parameters were recorded: gender, patient age at the time the PR was taken, and the date of the PR recording.

### Panoramic radiograph examination

The PRs were viewed and assessed by a previously calibrated investigator (RT). The calibration was carried out by "self-education" using 10 selected PR pictures for which the findings had been established (5 with/5 without carotid calcification). The five PRs with putative positive findings of carotid calcification were also assessed by a medical radiologist. Three cases were confirmed.

All PR pictures were examined one time. The examination of the PRs took place under standardized conditions in a darkened room on an x-ray viewer wit darkening device (light box).

At the beginning of the investigation, the corresponding PRs were examined in terms of possible carotid calcifications (suspected diagnosis) on the right side, left side or on both sides.

In addition, the number of missing teeth (tooth loss, the reason for tooth loss was unknown), as well as a assessment of (max) bone loss were documented. For this purpose, accordingly, in the lateral tooth area, the greatest bone destruction, the root length (distance from the enamel-cement-junction (ECJ) to the tip of the root (in mm), as well as bone loss: distance of the enamel-cement-junction (ECJ) to the limbus alveolaris (LA) in millimeter (mm), were measured using a millimeter-scaled periodontal probe (PCP 15, Hu-Friedy, Chicago, US). Then the calculation of the degree of bone loss was carried out by dividing the maximum bone loss (mm) by the root length (mm) and converting this figure to a percentage. Patients without posterior teeth were assigned a root length of 1 mm, bone loss of 1 mm and so a bone reduction of 100%. By dividing the bone reduction by the age of the patient at the time the radiograph was taken, the bone loss/age-index could be calculated.

Based on the parameter number of missing teeth and bone loss/age index, following of concept of Lang and Tonetti [30], an estimate of the periodontal risk was made in a modified form:

➣ low risk: number of missing teeth ≤ 4, bone reduction index ≤ 0.5,

➣ moderate risk: number of missing teeth 5 to 8 and/or bone reduction index 0.51-1.0,

➣ high risk: number of missing teeth ≥ 9 and/or bone reduction index ≥ 1.1;

The most unfavorable factor determined the overall degree of risk.

### Statistical analysis

The statistical analysis was carried out with the aid of SPSS for Windows, Version 15.0 (SPSS Inc., USA). The continuous variables were assessed in terms of their normal distribution using the Kolmogorov-Smirnov test. For comparing samples (carotid calcification: yes or no), non-parametric tests were always used. When comparing two independent, non-normally distributed samples, the Mann-Whitney U test was applied (age, tooth loss). The categorized data were analyzed with the aid of the Chi-square test or Fisher's exact test (gender, periodontal risk). A two-sided significance test was made of all tests and, for all statistical analysis a value of p < 0.05 was taken as being significant. In addition, a multivariate logistic model (binary logistic regression) for the association of carotid calcification with following parameters was performed: gender, age, tooth loss, periodontal risk, max. bone loss.

## Results

### Subjects

In total, 824 PRs (= 100%) from the patient population (54%) were included in the study. Of these, 349 were male (42.4%), and 475 were female (57.6%); the mean age of the patients at the time of the panoramic radiograph imaging was 48.32 ± 16.52 years (Table 1). The difference in age between the two genders (male: 47.67 ± 15.32, female: 48.8 ± 17.35) at the time the radiograph were taken was not significant (p = 0.45) (Table 1).

**Table 1:** Patient characteristics: Mean age (± sd), gender, mean teeth loss (± sd), periodontal risk, and suspected carotid calcification

Factor		
**age in years (mean ± sd)**		48.32 ± 16.52
**gender (n = 824)**	**female**	n = 475
	**male**	n = 349
**tooth loss (mean ± sd)**		4.16 ± 5.39
**periodontal risk**	**low**	n = 282
	**moderate**	n = 335
	**high**	n = 107
**carotid calcification**	**all**	n = 74 (9%)
	**right sides**	n = 45 (5.5%)
	**left sides**	n = 19 (2.3%)
	**both sides**	n = 10 (1.2%)

(sd: standard deviation)

### Panoramic radiograph examination

#### Carotid calcification

Suspected carotid calcification was determined in a total of 9% of the patients (n = 74). In 45 patients (5.5%), the carotid calcification was on the right side, in 19 patients (2.3%) on the left side, and in 10 patients on both sides (1.2%). In the remaining 750 patients (91.0%), no carotid calcification was found (Table 1).

#### Tooth loss

The mean tooth loss was 4.16 ± 5.39 (min: 0; max 28); so that, on average, the patients still had 23-24 teeth (Table 1). 259 patients still had all of their teeth, and 10 patients were toothless.

#### Periodontal risk

In accordance with the definition used, 282 patients (34.2%) had a low risk, 335 patients (40.7%) had a moderate risk, and 207 patients (25.1%) had a high risk of periodontitis (Table 1).

### Distribution of carotid calcification according to age, gender, loss of teeth, and periodontal risk

#### Age

The 750 patients in whom carotid calcification was not suspected were, on average, 46.94 ± 16.14 years old, whereas the 74 patients in whom carotid calcification was detected were, on average, 62.34 ± 13.70 years old. The difference was significant (p < 0.001) (Table 2).

**Table 2:** Distribution of age, gender, tooth loss, and periodontal risk according to diagnosis of suspected carotid calcification and level of significance (p < 0

		carotid calcification		significance
Factor		yes (n = 74)	no (n = 750)	(p < 0, 05)
**age in years (mean ± sd)**		62.34 ± 13.70	46.94 ± 16.14	p < 0.0001
**gender**	**female**	n = 50 (11%)	n = 425 (89%)	p = 0.07
	**male**	n = 24 (7%)	n = 325 (93%)	
**tooth loss**		7.62 ± 7.05	3.81 ± 5.08	p < 0.0001
**periodontal** **risk**	**low **(n = 282)	n = 9 (3.2%//12.2%)	n = 273 (96.8%//36.4%)	p < 0.0001
	**moderate **(n = 335)	n = 33 (9.9%//44.6%)	n = 302 (90.1%//40.3%)	
	**high **(n = 107)	n = 32 (15.5%//43.2%)	n = 175 (84.5%//23.3%)	

(sd: standard deviation)

#### Gender

In 24 (6.9%) out of 349 male and 50 (10.5%) out of 475 female subjects, carotid calcification was suspected. Accordingly, there was a tendency in the female subjects to have a higher prevalence of carotid calcification than the male patients, but the difference was, however, not significant (p = 0.084) (Table 2).

#### Tooth loss

Patients with no suspected carotid calcification had a mean tooth loss of 3.81 ± 5.08. Patients with a suspected carotid calcification had 7.62 ± 7.05 missing teeth. The difference was significant (p < 0.0001) (Table 2).

#### Risk of periodontitis

Of the 282 patients with a low risk of periodontitis, 9 (3.2%) had suspected carotid calcification (12.2% of all suspected cases). In patients with a moderate risk of periodontitis (n = 335), carotid calcification was suspected in 33 subjects (9.9%), and in subjects with a high risk of periodontitis (n = 207), this value was 32 (15.5%). These are 44.6% or 43.2%, respectively, of all cases of suspected carotid calcification. The influence of a risk of periodontitis was significant (p < 0.0001) (Table 2).

In the multivariate logistic model, only age showed a significant association to carotid calcification (p < 0.0001) with an odds ratio (OR) of 4.9 (confidence intervall [CI]: 2.4 - 9.8). The periodontal risk showed no significant association (p = 0.12) with an OR 1.4 (CI: 0.9 - 2.4). The results of the multivariate logistic model are given in Table 3.

**Table 3:** Multivariate logistic model for association of carotid calcification with following parameters: gender, age, tooth loss, periodontal risk, max. bone loss

Parameter	regressions coefficient	standard failure	significance (p - value)	odds ratio (OR)	95% confidence intervall
**gender**	0.47	0.27	0.08	1.61	0.95 - 2.7
**age**	1.59	0.36	0.000	4.88	2.4 - 9.8
**tooth loss**	0.02	0.03	0.39	1.02	0.97 - 1.1
**periodontal risk**	0.38	0.25	0.12	1.47	0.9 - 2.4
**max. bone loss**	0.001	0.01	0.93	1.00	0.99 - 1.0

## Discussion

In the present study, the prevalence of the diagnosis of suspected carotid calcification was found to be 9.0%. At the same time, the right side, with a value of 5.5%, had prevalence twice that for the left side (2.3%). The majority of the patients investigated (65%) had a moderate to high risk of periodontitis; on average, there were 4.16 ± 5.39 teeth missing. In addition, it was possible to establish a significant correlation between cases of suspected calcification with the risk of periodontitis, the number of missing teeth, and the age of the patients. However, only age showed a significant association (OR: 4.9; p < 0.0001).

In the international literature, depending on the study, prevalence of carotid calcification in PRs were found to be between 0.4 and 4.0% [5,31-37]. The large range of values given for prevalence of positive findings of carotid calcification in PRs could be explained by the different age groups, different distributions of gender, ethnicity and lifestyles of the particular patients investigated [5]. In the present study, the prevalence rate of 9% is clearly higher than that found in comparable studies and, at the same time, calcification was found both unilaterally and bilaterally - on the right side more frequently than on the left side or on both sides. Other studies have also reported an increased appearance of unilateral carotid calcification on the right side [5,38], with Ariayi et al. reporting that most positive findings were found bilaterally [5]. In contrast, Ohba et al. reported that most calcification of the arteria carotis lay on the left side [35]. In addition, women appear to be more frequently affected than men [5,36]. In the present study, women were also more frequently affected. The difference, however, was not significant, i.e. gender showed no significant association to carotid calcification (OR: 1.6). Another study revealed no influence of gender, too [39].

One explanation for the increased prevalence of a diagnosis of suspected carotid calcification in this study compared to others could be false-positive findings. It cannot be excluded that plaque from the arteria carotis is mistaken as calcification in the cartilago triticea, thereby resulting in possible false-positive findings [40,41]. Also, it should be considered that the study was carried out by a dentist from the practice who indeed was calibrated before, but not however by a specialist trained in radiology and a corresponding qualification. The results of one study regarding the effectiveness of a training program for the detection of carotid calcification in dental PRs revealed that only approximately 1/3 of the carotid calcifications identified by two trained dentists were also confirmed by a radiologist specializing in mouth, jaw and facial radiology [42]. Likewise, it can be assumed that of the 9.0% of suspected cases of carotid calcification in the study presented here, only about 3% would be confirmed findings. Therefore, it would seem that the prevalence of suspected cases of carotid calcification in our study is similar to that in comparable studies. However, it is quite conceivable that the diagnosed carotid calcifications in PRs show true calcification more precisely. In some studies, positive Doppler sonography results for the arteria carotis have been verified using conventional PR [43,44]. In other studies, on the one hand, 34.7% of carotid calcifications found in dental PRs were also found with ultrasound [39]; on the other hand, 62.3% were confirmed by CTs. The sensitivity was 22.2% and the specificity 90% [45].

In contrast, Tanaka et al. reported the ineffectiveness of findings of suspected carotid calcification in dental panorama tomography for assessing the risk of vessel disease and their further development to the point of death in 80-year-old patients [12]. However, one should critically question whether results for 80-year-old patients can be unconditionally translated to the effectiveness of the procedure in the case of younger patients and the course of their illnesses. Accordingly, Friedländer et al. reported that 37% of patients with a cerebral insult starting from the arteria carotis showed a corresponding carotid calcification in the PR [7].

The results of the study presented here showed an age-dependent frequency of positive findings of carotid calcifications. The fact that the risk of a stroke and a heart attack increases with age is well known [46]. At the same time, from the 50th year of life on, the extent of arteriosclerosis of the arteria carotis appears to be closely correlated with coronary heart disease and is an important indicator for identifying patients with coronary heart disease [46]. The results of the study presented here therefore confirm that, with increasing age, there is also an increase in suspected cases of carotid calcification and an age-dependent risk of carotid calcification (OR: 4.9).

In addition, the significant correlation between an increased risk of periodontitis and the occurrence of suspected cases of carotid calcifications, as well as the number of missing teeth is noticeable. However, a significant association to carotid calcification could not be found (OR: 1.5; p > 0.05). Other studies have likewise shown that an increased loss of alveolar bone which, as a rule, is associated with a periodontal history is correlated with an increased occurrence of carotid calcification [8,43,47]. Corresponding to Engebretson et al., bone loss is associated with carotid calcification (OR: 3.6; CI:1.4 - 9.7) [47]; in this study the OR for max. bone loss was 1.0 (CI: 0.99 - 1.0). At the same time, confirmed positive findings in Doppler sonography are connected with a high probability of periodontal disease [48]. In addition, the thickness of the intermedia of the arteria carotis appears to be associated with the presence of periodontitis [48]. Several case-control studies and cross-sectional studies have confirmed the existence of an association between periodontitis and cardiovascular diseases [25,49-55]. Madianos et al. conclued in their review a risk ranged from 0 to 3.3-fold for the occurrence of periodontal disease and CHD or a significant association between these both diseases, repectively [56]. On the basis of different meta-analyses, the odds ratio of this association was stated by 1.1 to 2.2 [27-29]. In the present study the odds ratio was 1.5 (CI: 0.0 - 2.4). Christou et al. showed with a limited number of subjects (n = 15) that, in addition to high blood pressure, the presence of periodontal disease was significantly correlated with a positive finding of carotid calcification [57].

With a large subject population the results presented here confirm the close correlation between the two diseases and make clear the fact that an interdisciplinary concept is necessary in dental and medical diagnostics for the determination of the risk of the two diseases. Consequently, the identification of such changes in the PR can be viewed as a contribution on the part of dentistry to secondary (chance) findings precautionary diagnostics. At the same time, one should take into account and weigh up the fact that in healthcare, in addition to the sensitivity and specificity of an investigation method, it is important how frequent these examinations are actually used by the population. Since most of the population receives dental care, PRs are carried out with certain regularity. Therefore, it should be considered whether, as a result of the use of better radiological diagnostics and the recording of secondary (chance) findings in dental practices, benefits can be achieved for patients in terms of health and economic value.

Limitation of the study: This cross sectional study was an investigation exclusively carried out on the basis of available PRs: a supplementary clinical dental and internist examination of the patients was not done. Accordingly, a positive finding of carotid calcification was not verified by means of further diagnostics (CT, sonography). For this reason, the positive findings were only suspected cases of carotid calcification.

Likewise, the assessment of periodontal risk was only made with by means of radiologically determined parameters, such as missing teeth, and the bone reduction age index. These were selected based on the periodontal risk assessment concept of Lang and Tonetti [30] in a modified and reduced version for the assessment of periodontal history and judgment of further periodontal risk. Additional parameters, such as smoking and systemic factors, were not used for risk assessment, as these data were not available to the same extent for all patients or they were incomplete, respectively. Nevertheless, one should keep in mind that smoking is a "high" risk factor for periodontal disease and CHD and represents an important confounder [58-60]. Furthermore, other dental infections, e.g. periapical infections or active caries decay, are also infections of oral disease burden, however, these infections were not considered in this study.

## Conclusion

PR can, as an additional diagnostic tool, provide an indication of carotid calcification as secondary (chance) finding. An unambiguous exclusion of changes due to calcification is, however, not possible. In addition, age, tooth loss, and periodontal risk are closely correlated with positive findings of carotid calcification. However, only age showed a significant association with a increase risk for carotid calcification.

Considering the corresponding anamnesis and the establishment of further risk factors for arteriosclerosis, such as smoking and diabetes mellitus, in a patient, the dentist must carry out more precise diagnostics in relation to the presence of carotid calcifications in PR. Close communication with the internist responsible is a fundamental prerequisite for achieving the implementation of further diagnostics with sonography or CT.

## Competing interests

The authors declare that they have no competing interests.

## Authors' contributions

RT has made substantial contributions to conception and design of the study; he carried out the examination and performed the statistical analysis. WB was the head of the study and made substantial contributions to conception and design of the study. SR has been involved in revising it critically for important intellectual content and gaven final approval of the version to be published. DZ conceived of the study, and participated in its design and coordination, interpretation of data and wrote the manuscript.

All authors read and approved the final manuscript.

## Pre-publication history

The pre-publication history for this paper can be accessed here:

http://www.biomedcentral.com/1471-2261/11/67/prepub

## References

[B1] Menken M Munsat TL Toole JF The globel burden of disease study: implications for neurology Arch Neurol 2000 57 418 420 10.1001/archneur.57.3.418 10714674

[B2] Lopez AD Mathers CD Ezzati M Jamison DT Murray CJ Global and regional burden of disease and risk factors, 2001: systematic analysis of population health data Lancet 2006 367 1747 1757 10.1016/S0140-6736(06)68770-9 16731270

[B3] Odink AE van der Lugt A Hofman A Hunink MGM Breteler MMB Krestin GP Witteman JCM Association between calcification in the coronary arteries, aortic arch and carotid arteries: the Rotterdam study Atherosclerosis 2007 193 408 413 10.1016/j.atherosclerosis.2006.07.007 16919637

[B4] Wyman RA Fraizer MC Keevil JG Busse KL Aeschlimann SE Korcarz CE Ultrasounddetected carotid plaque as a screening tool for advanced subclinical atherosclerosis Am Heart J 2005 150 1081 1085 10.1016/j.ahj.2005.01.010 16291002

[B5] Ariayi AS Berndt D Lambrecht JT Panoramic radiography for diagnosis of soft tissue calcifications - an aid to identify stroke prone patients? Schweiz Monatsschr Zahnmed 2009 119 1009 1013 in German 19954131

[B6] Carter LC Tsimidis K Fabiano J Carotid calcifications on panoramic radiography identify an asymptomatic male patient at risk for stroke Oral Surg Oral Med Oral Pathol Oral Radiol Endod 1998 85 119 122 10.1016/S1079-2104(98)90409-7 9474626

[B7] Friedlander AH Manesh F Wasterlain CG Prevalence of detectable carotid artery calcifications on panoramic radiographs of recent stroke victims Oral Surg Oral Med Oral Pathol Oral Radiol Endod 1994 77 669 673 10.1016/0030-4220(94)90332-8 8065736

[B8] Beckstrom BW Horsley SH Scheetz JP Khan Z Silveira AM Clark SJ Greenwell H Farman AG Correlation between carotid area calcifications and periodontitis: a retrospective study of digital panoramic radiographic findings in pretreatment cancer patients Oral Surg Oral Med Oral Pathol Oral Radiol Endod 2007 103 359 366 10.1016/j.tripleo.2006.08.016 17145189

[B9] Uthman AT Al-Saffar A Prevalence in digital panoramic radiographs of carotid area calcification among Iraqi individuals with stroke-related disease Oral Surg Oral Med Oral Pathol Oral Radiol Endod 2008 105 68 73 10.1016/j.tripleo.2007.11.009 18329570

[B10] Sung EC Friedlander AH Kobashigawa JA The prevalence of calcified carotid atheromas on the panoramic radiographs of patients with dilated cardiomyopathy Oral Surg Oral Med Oral Pathol Oral Radiol Endod 2004 97 404 407 10.1016/j.tripleo.2003.08.025 15024368

[B11] Gokce C Sisman Y Sipahioglu M Ertas ET Akgunlu F Unal A Tokgoz B Oymak O Utas C The prevalence of carotid artery calcification on the panoramic radiographs of end-stage renal disease patients with peritoneal dialysis: do incidental findings provide life-saving information? J Int Med Res 2008 36 47 53 18230267 10.1177/147323000803600107

[B12] Tanaka T Morimoto Y Ansai T Okabe S Yamada K Tagushi A Awano S Kito S Takata Y Tkehara T Ohba T Can the presence of carotid artery calcification on panoramic radiographs predict the risk of vascular diseases among 80-year-olds? Oral Surg Oral Med Oral Pathol Oral Radiol Endod 2006 101 777 783 10.1016/j.tripleo.2005.10.035 16731400

[B13] Scannapieco FA Position paper of The American Academy of Periodontology: Periodontal disease as a potential risk factor for systemic diseases J Periodontol 1998 69 841 850 9706864

[B14] Mealey BL Oates TW Diabetes mellitus and periodontal diseases J Periodontol 2006 77 1289 1303 10.1902/jop.2006.050459 16881798

[B15] D'Aiuto F Graziani F Tetè S Gabriele M Tonetti MS Periodontitis: from local infection to systemic diseases Int J Immunopathol Pharmacol 2005 18 1 11 16848982

[B16] Seymour GJ Ford PJ Cullinan MP Leishman S Yamazaki K Relationship between periodontal infections and systemic disease Clin Microbiol Infect 2007 13 3 10 10.1111/j.1469-0691.2007.01798.x 17716290

[B17] Lockhart PB Brennan MT Thornhill M Michalowicz BS Noll J Bahrani-Mougeot FK Sasser HC Poor oral hygiene as a risk factor for infective endocarditis-related bacteremia J Am Dent Assoc 2009 140 1238 1244 19797553 10.14219/jada.archive.2009.0046 PMC2770162

[B18] Persson GR Persson RE Cardiovascular disease and periodontitis: an update on the associations and risk J Clin Periodontol 2008 35 362 79 18724863 10.1111/j.1600-051X.2008.01281.x

[B19] Tonetti MS Periodontitis and risk for atherosclerosis: an update on intervention trials J Clin Periodontol 2009 36 15 9 19432627 10.1111/j.1600-051X.2009.01417.x

[B20] Haraszthy VI Zambon JJ Trevisan M Zeid M Genco RJ Identification of periodontal pathogens in atheromatous plaques J Periodontol 2000 71 1554 1560 10.1902/jop.2000.71.10.1554 11063387

[B21] Doĝan B Buduneli E Emingil G Atilla G Akilli A Antinheimo J Lakio L Asikainen S Characteristics of periodontal microflora in acute myocardial infarction J Periodontol 2005 76 740 748 10.1902/jop.2005.76.5.740 15898935

[B22] Pitiphat W Joshipura KJ Gillman MW Williams PL Douglass CW Rich-Edwards JW Maternal periodontitis and adverse pregnancy outcomes Community Dent Oral Epidemiol 2008 36 3 11 18205634 10.1111/j.1600-0528.2006.00363.x

[B23] Socransky SS Haffajee AD Dental biofilms: difficult therapeutic targets Periodontol 2000 2002 28 12 55 10.1034/j.1600-0757.2002.280102.x 12013340

[B24] Dye BA Choudhary K Shea S Papapanou PN Serum antibodies to periodontal pathogens and markers of systemic inflammation J Clin Periodontol 2005 32 1189 99 10.1111/j.1600-051X.2005.00856.x 16268994

[B25] Dietrich T Jimenez M Krall Kaye EA Vokonas PS Garcia RI Age-dependent associations between chronic periodontitis/edentulism and risk of coronary heart disease Circulation 2008 117 1668 74 10.1161/CIRCULATIONAHA.107.711507 18362228 PMC2582144

[B26] Janket S Baird AE Chuang S Jones JA Meta analysis of periodontal disease and risk of coronary heart disease and stroke Oral Surg Oral Med Oral Pathol Oral Radiol Endod 2003 95 559 569 10.1067/moe.2003.107 12738947

[B27] Bahekar AA Singh S Saha S Molnar J Arora R The prevalence and incidence of coronary heart disease is significantly increased in periodontitis: a meta-analysis Am Heart J 2007 154 830 837 10.1016/j.ahj.2007.06.037 17967586

[B28] Mustapha IZ Debrey S Oladubu M Ugarte R Markers of systemic bacterial exposure in periodontal disease and cardiovascular disease risk: a systematic review and meta-analysis J Periodontol 2007 78 2289 2302 10.1902/jop.2007.070140 18052701

[B29] Humphrey LL Fu R Buckley DI Freeman M Helfand M Periodontal disease and coronary heart disease incidence: a systematic review and meta-analysis J Gen Intern Med 2008 23 2079 2086 10.1007/s11606-008-0787-6 18807098 PMC2596495

[B30] Lang NP Tonetti MS Periodontal risk assessment (PRA) for patients in supportive periodontal therapy (SPT) Oral Health Prev Dent 2003 1 7 16 15643744

[B31] Kumagai M Yamagishi T Fukui N Chiba M Carotid artery calcification seen on panoramic dental radiographs in the Asian population in Japan Dentomaxillofac Radiol 2007 36 92 96 10.1259/dmfr/79378783 17403886

[B32] Lewis DA Brooks SL Carotid artery calcification in a general dental population: a retrospective study of panoramic radiographs Gen Dent 1999 54 19 103 10321159

[B33] Hubar JS Carotid artery calcification in the blackpolpulation: a retrospective study on panoramic radiographs Dentomaxillofac Radiol 1999 28 348 350 10.1038/sj.dmfr.4600478 10578188

[B34] Almog DM Carter LC Horev T Illig MD Green RM Correlating carotid artery stenosis detected by panoramic radiography with clinically relevant carotid artery stenosis determined by duplex ultrasound Oral Surg Oral Med Oral Pathol Oral Radiol Endod 2002 94 768 773 10.1067/moe.2002.128965 12464905

[B35] Ohba T Takata Y Ansai T Morimoto Y Tanaka T Kito S Awano S Akifusa S Takehara T Evaluation of calcified carotid artery atheromas detected by panoramic radiograph among 80-year-olds Oral Surg Oral Med Oral Pathol Oral Radiol Endod 2003 96 647 650 10.1016/j.tripleo.2003.07.001 14600703

[B36] Tamura T Inui M Nakamura S Nakase M Okumura K Tagawa T Clinicostatistical study of carotid calcifications on panoramic radiographs Oral Dis 2005 11 314 317 10.1111/j.1601-0825.2005.01125.x 16120119

[B37] Pornprasertsuk-Damrongsri S Thanakun S Carotid artery calcification detected on panoramic radiographs in a group of Thai population Oral Surg Oral Med Oral Pathol Oral Radiol Endod 2006 101 110 115 10.1016/j.tripleo.2005.04.002 16360615

[B38] Friedlander AH Altman L Carotid artery atheromasin postmenopausal women. Their prevalence on panoramic radiographs and their relationship to atherogenic risk factors J Am Dent Assoc 2001 132 1130 1136 11575022 10.14219/jada.archive.2001.0340

[B39] Bayram B Uckan S Acikgoz A Müderrisoglu H Aydinalp A Digital panoramic radiography: a reliable method to diagnose carotid artery atheromas? Dentomaxillofac Radiol 2006 35 266 270 10.1259/dmfr/50195822 16798924

[B40] Carter LC Discrimination between calcified triticeous cartilage and calcified carotid atheroma on panoramic radiography Oral Surg Oral Med Oral Pathol Oral Radiol Endod 2000 90 108 110 10.1067/moe.2000.106297 10884645

[B41] Kamikawa RS Pereira MF Fernandes A Meurer MI Study of the localization of radiopacities similar to calcified carotid atheroma by means of panoramic radiography Oral Surg Oral Med Oral Pathol Oral Radiol Endod 2006 101 374 378 10.1016/j.tripleo.2005.03.030 16504872

[B42] Almog DM Tsimidis K Moss ME Gottlieb RH Carter LC Evaluation of a training program for detection of carotid artery calcifications on panoramic radiographs Oral Surg Oral Med Oral Pathol Oral Radiol Endod 2000 90 111 117 10.1067/moe.2000.107056 10884646

[B43] Ravon NA Hollender LG McDonald V Persson GR Signs of carotid calcification from dental panoramic radiographs are in agreement with Doppler sonography results J Clin Periodontol 2003 30 1084 90 10.1046/j.0303-6979.2003.00427.x 15002895

[B44] Friedlander AH Garrett N R Chin EE Baker JD Ultrasonographic confirmation of carotid artery atheromas diagnosed via panoramic radiography J Am Dent Assoc 2005 136 635 640 15966651 10.14219/jada.archive.2005.0235

[B45] Yoon SJ Yoon W Kim OS Lee JS Kang BC Diagnostic accuracy of panoramic radiography in the detection of calcified carotid artery Dentomaxillofac Radiol 2008 37 104 108 10.1259/dmfr/86909790 18239037

[B46] Craven TE Ryu JE Espeland MA Kahl FR McKinney WM Toole JF McMahan MR Thompson CJ Heiss G Crouse JR Evaluation of the associations between carotid artery atherosclerosis and coronary artery stenosis Acase-control study Circulation 1990 82 1230 1242 10.1161/01.cir.82.4.1230 2205416

[B47] Engebretson SP Lamster IB Elkind MSV Rundek T Serman NJ Demmer RT Radiographics measures of chronic periodontitis and carotid artery plaque Stroke 2005 36 561 566 10.1161/01.STR.0000155734.34652.6c 15692118 PMC2692923

[B48] Beck JD Elter JR Heiss G Couper D Mauriello SM Offenbacher S Relationship of periodontal disease to carotid artery intima-media wall thickness: the atherosclerosis risk in communities (ARIC) study Arterioscler Thromb Vasc Biol 2001 21 1816 1822 10.1161/hq1101.097803 11701471

[B49] DeStefano F Anda RF Kahn HS Williamson DF Russell CM Dental disease and risk of coronary heart disease and mortality Br Med J 1993 306 688 691 10.1136/bmj.306.6879.688 PMC1677081 8471920

[B50] Beck J Garcia R Heiss G Vokonas P Offenbacher S Periodontal disease and cardiovascular disease J Periodontol 1996 67 1123 1137 8910831 10.1902/jop.1996.67.10s.1123

[B51] Mattila KJ Asikainen S Wolf J Jousimies-Somer H Valtonen V Nieminen M Age, dental infections, and coronary heart disease J Dent Res 2000 79 756 760 10.1177/00220345000790020901 10728977

[B52] Katz J Chaushu G Sharabi Y On the association between hypercholesterolemia, cardiovacular disease and severe periodontal disease J Clin Periodontol 2001 28 865 868 10.1034/j.1600-051x.2001.028009865.x 11493357

[B53] Lopez R Oyarzun M Naranjo C Cumsille F Ortiz M Baelum V Coronary heart disease and periodontitis - a case control study in Chilean adults J Clin Periodontol 2002 29 468 473 10.1034/j.1600-051X.2002.290513.x 12060431

[B54] Persson RE Hollender LG Powell VL MacEntee M Wyatt CC Kiyak HA Persson GR Assessment of periodontal conditions and systemic disease in older subjects II. Focus on cardiovascular diseases. J Clin Periodontol 2002 29 803 810 10.1034/j.1600-051x.2002.290903.x 12423292

[B55] Hujoel PP Drangsholt M Spiekerman C DeRouen TA Pre-existing cardiovascular disease and periodontitis: a follow up study J Dent Res 2002 81 186 191 10.1177/154405910208100309 11876273

[B56] Madianos PN Bobetsis GA Kinane DF Is periodontitis associated with an increased risk of coronary heart disease and preterm and/or low birth weight births? J Clin Periodontol 2002 29 22 38 12787204 10.1034/j.1600-051x.29.s3.2.x

[B57] Christou P Leemann B Schimmel M Kiliaridis S Müller F Carotid artery calcification in ischemic stroke patients detected in standard dental panoramic radiographs - a preliminary study Adv Med Sci 2010 55 26 31 10.2478/v10039-010-0022-7 20513642

[B58] Heasman L Stacey F Preshaw PM McCracken GI Hepburn S Heasman PA The effect of smoking on periodontal treatment response: a review of clinical evidence J Clin Periodontol 2006 33 241 253 10.1111/j.1600-051X.2006.00902.x 16553633

[B59] Erhardt L Cigarette smoking: an untreated risk factor for cardiovascular disease Atherosclerosis 2009 205 23 32 10.1016/j.atherosclerosis.2009.01.007 19217623

[B60] Hujoel PP Drangsholt M Spiekerman C DeRouen TA Periodontitis-systemic disease associations in the presence of smoking - causal or coincidental? Periodontol 2000 2002 30 51 60 10.1034/j.1600-0757.2002.03005.x 12236895

